# UNCIVILIZED CHILDREN OR VICTIMS OF DEMENTIA: INTERPRETATIONS OF AGGRESSION IN ASSISTED LIVING

**DOI:** 10.1093/geroni/igz038.1582

**Published:** 2019-11-08

**Authors:** Laura M Funk, Rachel Herron, Dale Spencer

**Affiliations:** 1 University of Manitoba, Winnipeg, Manitoba, Canada; 2 Brandon University, Manitoba, Canada; 3 Carleton University, Ottawa, Ontario, Canada

## Abstract

There is increasing interest in examining aggression in older adults and persons living with dementia (PLWD) in nursing homes. Relational or indirect aggression between tenants is also of concern in assisted living (A/L). However, there is a dearth of knowledge about how people interpret the meaning of aggression in older adults in A/L. Such interpretations inform responses within interactions as well as in management, practice and policy. This study explored interpretations of aggression in older tenants of A/L through thematic and narrative analyses of qualitative interview data from 32 participants: 13 tenants and 19 staff. Tenants downplayed the existence of ‘bullies’ but spoke of (or themselves enacted, in the interview) relational aggression in tenant conflicts with ‘troublemakers,’ complainers, slackers and PLWD. Dementia was not universally interpreted as excusing inappropriate or aggressive behaviours, and tenants expressed discomfort interacting with PLWD. The narratives of both groups (tenants and workers) drew on and reproduced stigmatizing and/or patronizing views of dementia and aging. Workers commonly positioned dementia (or other conditions such as physical illness, changes in routine, isolation, and loss of independence) as mitigating culpability for aggression. They did so in part to avoid taking aggression personally; some were also concerned about protecting tenants from disciplinary measures. Narratives about aggression reflect and further reinforce relations of power within A/L, with implications for everyday interactions of life and work in these settings. A/L facilities can address relational aggression in part through addressing talk and actions that perpetuate ageism and dementiaism.

